# Echocardiographic Assessment in Various Obesity Phenotypes: A Cross-Sectional Study

**DOI:** 10.7759/cureus.78716

**Published:** 2025-02-07

**Authors:** Vankadari Venkata Sesha Satya Sagar, Sourya Acharya, Sunil Kumar, Harshitha Reddy, Roma Chavhan, Nikhil Reddy

**Affiliations:** 1 Internal Medicine, Jawaharlal Nehru Medical College, Datta Meghe Institute of Higher Education and Research, Wardha, IND

**Keywords:** echocardiography, insulin resistance, metabolic syndrome, obesity, syndrome x

## Abstract

Background

Obesity is a risk factor for metabolic syndrome, which is a combination of metabolic abnormalities leading to development of cardiovascular abnormalities. Based on factors such as body mass index and metabolic syndrome, specific phenotypes for obesity have been established. These include metabolically healthy obese (MHO), metabolically unhealthy non-obese (MUNO), metabolically unhealthy obese (MUO), and metabolically healthy non-obese (MHNO). Echocardiography is a standard, noninvasive modality that is widely used to assess cardiovascular function. A systematic review and meta-analysis of echocardiographic studies in adult obesity found that obese adults were 4.2 times more likely to have left ventricular hypertrophy than nonobese adults.This study was conducted with the aim of the echocardiographic assessment of cardiac function in various obesity phenotypes.

Material and methods

This observational study was done in a tertiary care hospital and conducted for a period of two years from August 2019 to August 2021. Anthropometric data was obtained and metabolic parameters were estimated. After obtaining institutional ethical clearance, 400 patients were categorized into four groups of 100 based on their obesity phenotypes: MUO, MHO, MUNO, and 100 age- and sex-matched non-obese metabolically healthy individuals (MHNO) as controls. Echocardiographic assessment such as systolic and diastolic dysfunction was studied among above mentioned obesity phenotypes. The data was analysed using appropriate statistical significance tests.

Results

The mean BMI was highest in the MUO group (30.07 ±2.53), followed by MHO (28.79±2.3), and lowest in the MHNO group (22.77±1.13). The proportion of patients with Grade II diastolic dysfunction was higher in MUO patients (43%) compared to MHO patients (12%) and MUNO patients (16%). In contrast, the proportion of patients with Grade I diastolic dysfunction was lower in MUO patients (46%) compared to MHO patients (55%) and MUNO patients (57%). Systolic dysfunction in metabolically healthy non-obese (MHNO) patients (57.97 ± 2.34) was significantly higher than in MHO patients (51.83 ± 4.66, p < 0.0001), MUNO patients (51.49 ± 4.64, p < 0.0001), and MUO patients (49.9 ± 3.65, p < 0.0001).

The proportion of patients with Grade II diastolic dysfunction was higher in MUO (43%) compared to MHO (12%) and MUNO (16%). In contrast, the proportion of patients with Grade I diastolic dysfunction was lower in MUO (46%) when compared to MHO (55%) and MUNO (57%). Systolic dysfunction in MHNO (57.97±2.34) was significantly higher as compared to MHO (51.83±4.66, p-value<.0001), MUNO (51.49±4.64, p-value<0.0001) and MUO (49.9±3.65, p-value<0.0001).

Conclusion

Cardiac function abnormalities in various phenotypes exhibit a significant positive correlation, including ventricular systolic and diastolic dysfunctions. Therefore, multidisciplinary management of all obesity phenotypes should be initiated as early as possible to prevent future cardiovascular morbidity and mortality.

## Introduction

Obesity is defined as a state of abnormal or excessive accumulation of fat. It is usually assessed in clinical practice by the body mass index (BMI) obesity. Asian Indians were given revised guidelines about the diagnosis of obesity and metabolic syndrome to have a deceleration effect on the rising problems of diabetes and cardiovascular disease [1]. The major features of metabolic syndrome (MS) include abdominal obesity, elevated triglycerides (TG), lower high-density lipoprotein (HDL) cholesterol, rise in fasting blood sugar (FBS) levels, and rise in blood pressure. Even though obesity is the major risk factor for metabolic syndrome, subjects with normal weight may also be insulin resistant and may have metabolic syndrome [2]. Resistance may be symptomless, or a range of diseases may arise, such as Impairment of glucose metabolism, diabetes type II, hyperlipidemia, obesity, hypertriglyceridemia, and hypertension [3]. Insulin operates such that, on the one hand, all obese subjects may not have metabolic risk factors; on the other hand, all normal-weight subjects are not metabolically healthy. Obesity phenotypes have been classified based on metabolic syndrome parameters, including metabolically healthy obese (MHO), metabolically unhealthy non-obese (MUNO), metabolically unhealthy obese (MUO), and metabolically healthy non-obese (MHNO) [4-6]. Obesity rates in India are on the rise; while it was initially observed in urban areas and metro cities, it is now extending to smaller rural regions as well. Obesity contributes to a higher risk of cardiovascular abnormalities. Asian Indians exhibit unique features of obesity: excess body fat, abdominal adiposity, increased subcutaneous and intra-abdominal fat, and deposition of fat in ectopic sites like liver, muscle, etc. Based on wide consensus among experts, the criteria for obesity for the Asian Indian population proposed were BMI between 23 and 24.9 kg/m2, defined as overweight and 25 kg/m2 or more, as obesity and waist circumference (WC) of 80 cm or more in women, and 90 cm or more in men was defined as abdominal obesity [7].

Appropriate cardiac rehabilitation and lifestyle modifications can reduce the prevalence of metabolic syndrome. Recent echocardiographic modalities are sensitive in detecting subtle cardiac dysfunction. Systolic dysfunction can be detected before left ventricular ejection fraction (LVEF) changes by speckle tracking echocardiography through measurement of global longitudinal strain (GLS), hence easing the prediction of heart failure in asymptomatic subjects [7]. Obesity also causes dilatation and hypertrophy of the left ventricle, ultimately leading to diastolic dysfunction and worsening of left ventricular strain even with normal or mildly impaired LVEF. Left ventricular hypertrophy is also a common condition that profoundly affects morbidity and mortality in coronary artery disease, congestive cardiac failure, ventricular arrhythmia, and stroke [8]. Long-term high blood pressure can cause coronary artery disease, stroke, heart failure, peripheral vascular disease, vision loss, and chronic kidney disease. Left ventricular hypertrophy, considered to be one of the parts of target organ damage, has been used as one of the markers of long-term hypertension [1]. Thus, the present study was conducted to determine the echocardiographic changes concerning obesity phenotypes.

## Materials and methods

The observational study was conducted over two years, from August 2019 to August 2021, in the General Medicine Department at Acharya Vinobha Bhave Rural Hospital in Jawaharlal Nehru Medical College, Wardha, India. The study commenced after approval from the Institutional Ethics Committee, with approval number DMIMS(DU)/IEC/Aug-2019/8221. Informed consent was taken from the participants who comprised patients visiting the outpatient department, as well as patients admitted to the hospital. We also included medical students and paramedical staff of our university above the age of 18. Patients on long-term corticosteroid therapy, chronic renal failure, chronic liver failure, valvular heart disease, coronary artery disease, and heart failure were excluded. Detailed physical examination and anthropometric measurements in weight, height, body mass index (BMI), waist circumference (WC), systolic blood pressure (SBP), and diastolic blood pressure (DBP) were calculated. Biochemistry analysis, including FBS, TG, and HDL, was estimated. All the anthropometric measurements were taken on the day of admission which were taken by an independent observer who was blind to the study.

Sample size

The sample size was calculated by using the formula:

$n= Z 2 &alpha;/2 X P X (1-P)/d 2$

Where Zα/2 represents the significance level at 5% (95% confidence interval [CI] = 1.96), P is the prevalence of obesity (12.5% or 0.125), d is the derived margin of error (7% or 0.07), and n is the patients needed in each group (n=90). Approximately 100 patients were included in the study in each group. The total number of patients was set at 100 in each group for convenience, despite the sample size formula suggesting 90. While this exceeded the calculated requirement, it was maintained to enhance statistical power. The prevalence of 12.5% was selected according to previous studies reporting the proportion of individuals with the same disease. The larger margin of error is 7% usually acceptable for exploratory or feasible studies where resources or time are limited.

Anthropometric features were measured, including weight, height, BMI, and waist circumference. Weight in kilograms was checked standing motionless on the standard weighing machine on bare feet and simple clothing. The WC in centimetres was checked with the help of a non-stretchable measuring tape placed equidistant between the last rib palpated and the upper border of the iliac crest in normal exhalation in the erect posture. Heavy clothing in the waist region was removed before the measurement. Under aseptic precautions, a venous blood sample was collected accordingly in the plain bulb and fluoride bulb. The sample was immediately centrifuged for serum separation. Blood samples were analyzed by VITROS 5600 Dry Chemistry Autoanalyzer (QuidelOrtho Corporation., San Diego, CA) within one hour of collection to preserve biomarker stability. This autoanalyzer calibration involves loading bar-coded calibrators, generating calibration curves, and regularly running quality control samples to monitor system performance and ensure ongoing accuracy. Assay variability includes within run, between run and reagent lot differences influenced by instrument calibration.

Patients underwent echocardiographic assessment using the Philips HD11XE, 2-4MHZ linear probe (Philips, Amsterdam, Netherlands). Left ventricular systolic function was assessed using Simpson’s formula. Left ventricular end-diastolic volume (LV EDV) and left ventricular end-systolic volume (LV ESV) were quantified by tracing the inner borders of the left ventricle during diastole and systole in the apical four-chamber view. LVEF was calculated using the formula:

$(LV EDV - LV ESV) / LV EDV &times; 100$

Diastolic function was assessed by the values obtained by E/A, E/E, E-wave deceleration time, isovolumetric relaxation time and left atrial volume values and graded as normal or Grade I through Grade IV by the American Society of Echocardiography.

Study definition

Obesity in this study was defined as per the BMI categories for Asian Indians, revised based on consensus guidelines. The revised guidelines categorize obesity as a BMI ≥25 Kg/m2. MS was defined per the Modified National Cholesterol Education Programme Adult Treatment Panel III (NCEP ATP III) criteria. According to these BMI categories, a normal BMI is 18.0-22.9 kg/m², overweight is 23.0-24.9 kg/m², and obesity is ≥25 kg/m². The Modified NCEP ATP III criteria require the presence of at least three of the following components: a WC of ≥90 cm for South Asian men or ≥80 cm for South Asian women, TG ≥150 mg/dL, HDL cholesterol <40 mg/dL for men or <50 mg/dL for women, SBP/DBP ≥130/85 mmHg, and fasting plasma glucose ≥100 mg/dL [7].

Phenotypes

Metabolically unhealthy obese (MUO) were defined as those who were obese (BMI more than equal to 25kg/m2) and had metabolic syndrome, i.e., more than/equal to three MetS criteria as per modified National Cholesterol Education Program and Adult Treatment Panel-III (NCEP ATP III) criteria. Metabolically healthy obese (MHO) were defined as those who were obese (BMI more than equal to 25 kg/m2) and did not have metabolic syndrome, i.e., less than three MetS criteria as per modified NCEP ATP III criteria. Metabolically unhealthy nonobese (MUNO) was defined as nonobese (BMI less than 25 kg/m2) subjects with metabolic syndrome, i.e., more than/ equal to three MetS criteria as per modified NCEP ATP III criteria. Metabolically healthy nonobese (MHNO) were defined as nonobese (BMI less than 25kg/m2) and did not have metabolic syndrome, considered as controls in the current study.

Statistical analysis

The categorical variables were presented as numbers and percentages (%). On the other hand, the presentation of the continuous variables was done as mean ± SD and median values. The comparison of the variables which they were quantitative and were analyzed using ANOVA (for normally distributed data), and a post hoc test (Bonferroni correction) was applied. The comparison of the qualitative variables was analyzed using the chi-square test. If any cell had an expected value of less than 5, then Fisher’s exact test was used. The data was recorded in a Microsoft Excel spreadsheet (Microsoft Corp., Redmond, WA), and the final analysis was done using Statistical Package for Social Sciences (SPSS) software, ver 21.0 (IBM Corp., Armonk, NY). A p-value of less than 0.05 was considered significant.

## Results

A total of 400 patients were included in the study, consisting of 100 with MUO, 100 with MHO, 100 with MUNO, and 100 age- and sex-matched MHNO as controls. The mean age was similar in all the four groups. The MUNO and MHNO groups had patients with normal BMI who were overweight, whereas all the obese patients were grouped into MHO and MUO groups. The mean BMI was highest in the MUO group (30.07 ±2.53), followed by the MHO group (28.79 ± 2.3), and lowest in the MHNO group (22.71 ±1.16). All other baseline characteristics have been highlighted in Table 1. 

**Table 1:** Comparison of demographic characteristics between different phenotypes ^† ^chi-square test; ^‡^ ANOVA MHO: metabolically healthy obese; MUNO: metabolically unhealthy non obese; MUO: metabolically unhealthy obese; MHNO: metabolically healthy non obese; SD: standard deviation; BMI: body mass index; WC: waist circumference; cm: centimeter; SBP: systolic blood pressure; DBP: diastolic blood pressure; mmHg: millimeter of mercury; FBS: fasting blood sugar; mg/dL: milligram per decilitre; TG: triglycerides; HDL: high density lipoprotein

Demographic characteristic		MHO (n=100)	MUNO (n=100)	MUO (n=100)	MHNO (n=100)	P-value
Age (years)	Mean ± SD	53.64 ± 14.79	54.54 ± 13.66	56.43 ±11.48	54.59 ± 13.2	<0.511 ^‡^
Gender	Female	30 (30%)	34 (34%)	31 (31%)	34 (34%)	<0.9^†^
	Male	70 (70%)	66 (66%)	69 (69%)	66 (66%)	
BMI	18-22.9 (normal BMI)	0 (0%)	57(57%)	0 (0%)	63 (63%)	<0.0001
	23-24.99 (overweight)	0 (0%)	43 (43%)	0 (0%)	37 (37%)	
	>=25 (obese)	100 (100%)	0 (0%)	100 (100%)	0 (0%)	
	Mean ± SD	28.79 ± 2.3	22.71 ± 1.16	30.07 ± 2.53	22.77 ± 1.13	< 0.0001
WC (cm)	Deranged	49 (49%)	42 (42%)	76 (76%)	15 (15%)	< 0.0001
	Normal	51 (51%)	58 (58%)	24 (24%)	85 (85%)	
	Mean ± SD	84.47 ± 6.54	84.43 ± 6.53	97.48 ± 9.07	83.66 ± 5.02	< 0.0001
SBP (mmHg)	Mean ± SD	120.9 ± 14.15	130.14 ± 14.28	128.82 ± 13.61	115.3 ± 5.02	< 0.0001
DBP (mmHg)	Mean ± SD	79.5 ± 9.36	86.1 ± 9.2	84.8 ± 7.72	75.4 ± 5.01	< 0.0001
FBS (mg/dL)	Deranged	26 (26%)	78 (78%)	67 (67%)	2 (2%)	< 0.0001
	Normal	74 (74%)	22 (22%)	33 (33%)	98 (98%)	
	Mean ± SD	99.94 ± 29.49	130.02 ± 57.24	121.55 ± 54.58	88.24 ± 6.56	< 0.0001
TG	Deranged	23 (23%)	68 (68%)	77(77%)	14 (14%)	< 0.0001
	Normal	77 (77%)	32 (32%)	23 (23%)	86 (86%)	
	Mean ± SD	127.8 ± 36.32	165.03 ± 57.56	166.3 ± 56.12	126.57 ± 17.71	
HDL	Deranged	52 (52%)	69 (69%)	74 (74%)	30 (30%)	<0.0001 ^†^
	Normal	48 (48%)	31 (31%)	26 (26%)	70 (70%)	
	Mean ± SD	38.14 ± 9.4	34.86 ± 13.08	34.74 ± 10.94	42.80 ± 6.69	

**Figure 1:**
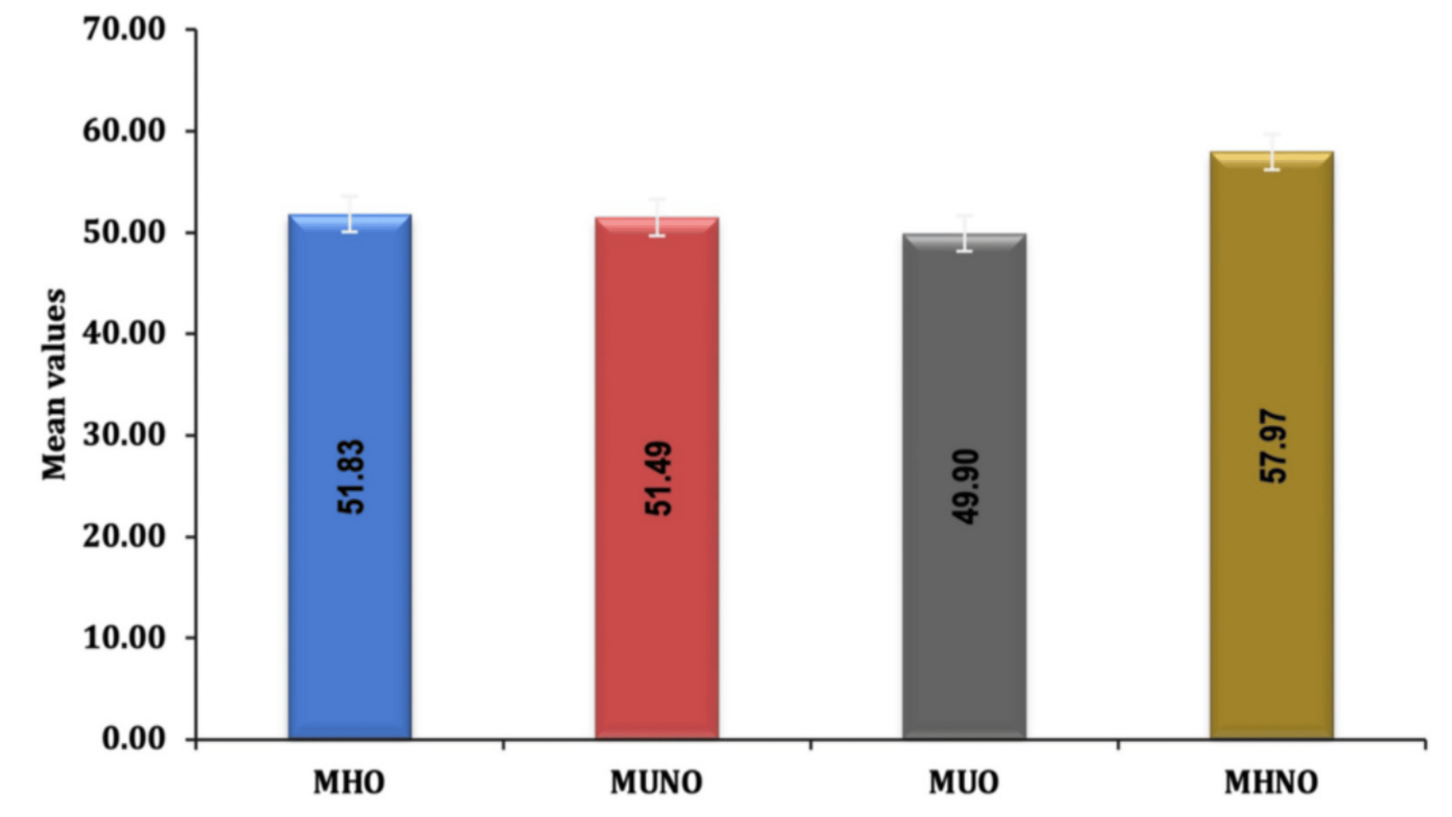
Comparison of LV systolic function (LVEF%) between different phenotypes MHO: metabolically healthy obese; MUNO: metabolically unhealthy non-obese; MUO: metabolically unhealthy obese; MHNO: metabolically healthy non-obese

Mean ± SD of LVEF (%) in MHNO (57.97 ± 2.34) was significantly higher as compared to MHO (51.83 ± 4.66, p-value<0.0001), MUNO (51.49 ± 4.64, p-value<0.0001) and MUO (49.9 ± 3.65, p-value<0.0001). The mean ± SD of LVEF (%) in MUO was significantly lower as compared to MHO (p-value<0.004) and MUNO (p-value<0.027). LVEF(%) was comparable between MHO and MUNO (p-value<1), as shown in Figure 1.

**Figure 2:**
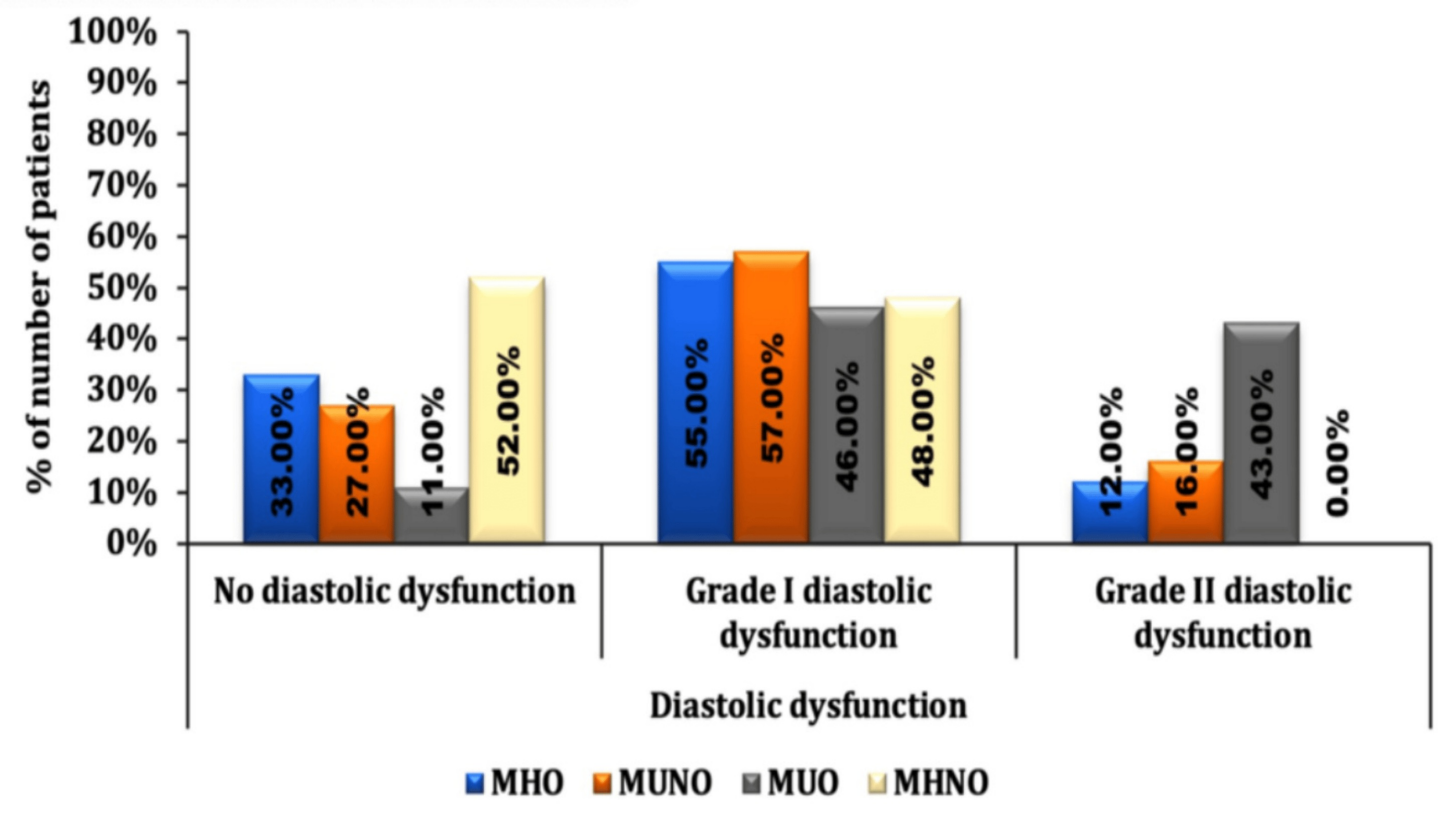
Comparison of diastolic dysfunction between different phenotypes MHO: metabolically healthy obese; MUNO: metabolically unhealthy non-obese; MUO: metabolically unhealthy obese; MHNO: metabolically healthy non-obese

The mean LVEF (%) was significantly lower in the MUO group (49.9 ± 3.65) compared to the MHO group (51.83 ± 4.66) and the MUNO group (51.49 ± 4.64), suggesting the combined impact of metabolic health and obesity in lowering the ejection fraction. The proportion of patients with Grade II diastolic dysfunction was higher in the MUO group (n=43; 43%) compared to MHO (n=12; 12%) and MUNO (n=16; 16%). The distribution of diastolic dysfunction was comparable between MHO and MUNO (p-value<0.547). The proportion of patients with no diastolic dysfunction or grade I diastolic dysfunction was significantly lower in MUO compared to MHO (p < 0.0001) and MUNO (p < 0.0001). The proportion of patients without diastolic dysfunction was significantly higher in MHNO (52%) as compared to MHO (33%, p-value<0.0002), MUNO (27%, p-value<0.0001), and MUO (11%, p-value<0.0001). In contrast, the proportion of patients with Grade I diastolic dysfunction was lower in MUO 46 (46%) when compared to MHO 55 (55%) and MUNO 57 (57%), suggestive of the combined impact of metabolic health and obesity on the severity of diastolic dysfunction as shown in Figure 2. 

By using multiple regression analysis, out of the seven variables that are correlated with systolic dysfunction, HDL, BMI, and FBS are significantly associated with systolic dysfunction, whereas TG, WC, SBP, and DBP are not correlated with systolic dysfunction, as shown in Table 2.

**Table 2:** Multiple logistic regression for systolic dysfunction TG: triglycerides; HDL: high density lipoprotein; BMI: body mass index; WC: waist circumference; SBP: systolic blood pressure; DBP: diastolic blood pressure; FBS: fasting blood sugar * these p-values are considered significant.

Variables		B	Std. Error	Wald	df	p-value	Exp (B)	95% confidence interval for Exp (B)	
								Lower Bound	Upper Bound
Systolic dysfunction	Intercept	-2.916	0.385						
	TG	0.4	0.263	2.3	1	0.129	1.491	0.89	2.499
	HDL	0.828	0.29	8.18	1	0.004*	2.289	1.298	4.039
	BMI	1.393	0.278	25.07	1	0.0001*	4.027	2.335	6.947
	WC	0.092	0.272	0.11	1	0.734	1.097	0.644	1.867
	SBP	1.987	1.303	2.32	1	0.127	7.292	0.567	6.378
	DBP	-0.109	1.296	0.007	1	0.933	0.897	0.071	1.138
	FBS	1.789	0.265	45.61	1	0.0001*	5.985	3.561	5.059

By using multiple regression analysis out of the seven variables correlated with diastolic dysfunction, HDL, BMI, and FBS are significantly correlated with diastolic dysfunction whereas TG, WC, SBP, and DBP are not correlated with diastolic dysfunction, as highlighted in Table 3.

**Table 3:** Multiple logistic regression for diastolic dysfunction TG: triglycerides; HDL: high density lipoprotein; BMI: body mass index; WC: waist circumference; SBP: systolic blood pressure; DBP: diastolic blood pressure; FBS: fasting blood sugar

Variables		B	Std. error	Wald	df	p-value	Exp (B)	95% confidence interval for Exp (B)	
								Lower Bound	Upper Bound
Diastolic dysfunction	Intercept	-3.627	0.514						
	TG	0.293	0.328	0.802	1	0.371	1.341	0.706	2.54
	HDL	1.068	0.381	7.861	1	0.005*	2.908	1.379	6.13
	BMI	2.371	0.354	44.938	1	0.0001*	10.709	5.354	7.41
	WC	0.446	0.325	1.877	1	0.171	1.562	0.825	2.95
	SBP	3.587	2.383	2.266	1	0.132	36.132	0.338	3.85
	DBP	0.089	2.369	0.001	1	0.97	1.093	0.011	1.13
	FBS	2.349	0.355	43.906	1	0.0001*	10.477	5.23	8.99

## Discussion

In this study, the mean age was similar across all four groups. However, MUO subjects were older than the other groups, with a mean age of 56.43 ± 11.48 years. Similar age distributions were observed in the studies conducted by Lee et al. [8], Wang et al. [9], and Dobson et al. [10]. However, in these studies, the highest mean age was found in the MUNW, MUOW, and MUNW groups, respectively [8,9,11].

Cultural differences shown in the following studies could also affect outcomes: Lee et al. [8] studied a population typically consuming a traditional diet rich in fermented foods, vegetables, and lower fat intake. On the other hand, Wang et al. examined a population experiencing dietary transitions due to urbanization, with increased consumption of processed foods and sugary beverages [9]. Efremov et al., whose study was based in Eastern Europe, studied a population exposed to high-fat, high-carbohydrate diets and different food environments [11].

Other methodological variations could be indicated based on the study population. Lee et al. included younger individuals, which could skew the prevalence of MUO [8]. Wang et al. focused on older populations or specific regions in China, potentially influencing the findings [9]. A study by Strait et al. found aging-related cardiovascular changes that could lead to heart failure in long-term obese phenotypes [12]. Individuals with MUO are often older due to age-related metabolic changes, cumulative lifestyle factors, changes in fat distribution, and chronic low-grade inflammation. These factors compound over time, increasing the risk of metabolic disorders as one age [13].

In this study, MUNO and MHNO included patients who had normal BMI and were overweight, whereas all the obese patients were grouped into MHO and MUO. The mean BMI was highest in the MUO group, followed by MHO, and lowest in the MHNO group. Similar findings were reported in studies by Lee et al. and Wang et al. [8,9], where BMI was highest in MUO individuals. In contrast, Efremov et al. [11] found the highest BMI in the MHO group. Studies by Dobson et al. [10] and Wang YC et al. [14] also observed higher BMI in obese phenotypes. Khan et al. [15] identified an association between BMI and cardiovascular changes; Yaylali et al. in their study found that BMI was a predictor for DD [16].

In this study, patients with deranged WC were significantly higher in MUO groups than in MHO, MUNO, and MHNO, showing that obesity contributes to increased WC. Among the obese groups (MUO, MHO), the mean WC was significantly higher in MUO group compared to MHO, suggesting central obesity due to metabolic derangement. In the studies conducted by Wang et al. and Efremov et al. [9,11], WC was highest in MHO and MUO groups. In studies by Dobson et al. [10] and Wang YC et al. [14], WC was highest in the obese phenotypes, the findings of which were similar to the current study.

In the present study, mean SBP was significantly higher in MUNO and MUO than in metabolically healthy groups (MHO, MHNO), suggesting a higher impact of metabolic derangement on SBP. SBP in MHO was significantly higher compared to MHNO (p value<0.009), suggesting an effect of obesity on SBP. Similar findings were seen in studies by Lee et al., Wang et al., and Efremov et al., [8,9,11] where SBP was higher in the metabolically unhealthy groups. In the study conducted by Dobson et al. [10] and Wang YC et al., [14] SBP was highest in the MHO group. Lee et al. also found that SBP greatly impacts cardiac dysfunction in metabolically unhealthy [8].

Mean DBP was significantly higher in MUNO and MUO groups than in metabolically healthy groups (MHO, MHNO), suggesting a higher impact of metabolic derangement on DBP. DBP in the MHO group was significantly higher compared to the MHNO group, suggesting the effects of obesity on DBP. In studies by Lee et al., Wang et al., and Efremov et al. [8,9,11], DBP was higher in the metabolically unhealthy groups. In the study conducted by Wang YC et al. [14] DBP was highest in the obese MS+ group. A possible explanation for this variation in systolic and DBP may be due to increased peripheral vascular resistance and endothelial dysfunction.

The proportion of patients with deranged FBS was significantly higher in MUNO and MUO compared to metabolically healthy groups (MHO and MHNO), suggesting a higher impact of metabolic health on FBS. A significantly higher proportion of patients had deranged FBS in MHO compared to MHNO, suggesting the contribution of obesity to the derangement of FBS. Mean FBS was significantly higher in the MUNO and MUO groups than in metabolically healthy groups (MHO and MHNO), suggesting a higher impact of metabolic health on FBS. Similar findings were obtained in the studies conducted by Lee et al. [8], Wang et al. [9], Efremov et al. [11], Dobson et al. [10], and Wang YC et al. [14] who found that fasting blood sugar levels are higher in the metabolically unhealthy groups when compared to the metabolically healthy groups.

The proportion of patients with deranged TGs was significantly higher in MUNO and MUO compared to metabolically healthy groups (MHO and MHNO), suggesting a higher impact of metabolic health on TGs. There is no significant difference between MHO and MHNO but between metabolically unhealthy and healthy groups, implying that metabolic health impacts TGs rather than obesity. Mean TG levels were significantly higher in MUNO and MUO than in metabolically healthy groups (MHO and MHNO), suggesting a higher impact of metabolic health on TGs. Similar findings were obtained in the studies conducted Lee et al., Wang et al., Efremov et al., Dobson et al., and Wang YC et al. [8,9,11,10,14] in which TG levels are higher in the metabolically unhealthy groups when compared to the metabolically healthy groups. Studies by Aberra et al [17]. In Ye et al. [18], TGs show independent risk with CVD.

The proportion of patients with deranged HDL was significantly higher in MUNO and MUO compared to metabolically healthy groups (MHO and MHNO), suggesting a higher impact of metabolic health on HDL. Distribution of deranged HDL was comparable between MHO and MHNO; hence obesity also impacts HDL other than metabolic health. Similar findings were obtained in the studies conducted by Lee et al. [8], Wang et al. [9], Efremov et al. [11], Dobson et al. [10], Wang et al. YC [14] wherein the proportion of patients with deranged HDL levels was higher in the metabolically unhealthy groups when compared to the metabolically healthy groups.

In the present study, mean LVEF was significantly lower in MUO compared to MHO and MUNO suggesting the combined impact of metabolic health and obesity in lowering the ejection fraction. Mean LVEF was highest in MHNO when compared to other groups. Mean LVEF was similar in MHO and MUNO, which was not statistically significant, implying metabolic health status and obesity have identical impacts on lowering ejection fraction in this study. Whereas mean LVEF in MHO and MUNO was significantly higher when compared to MHNO which implies that isolated obesity or metabolic derangement also have an impact in lowering LVEF. Similar findings were obtained in the study conducted by Wang et al. [9] in which LVEF was lower in the MUO group when compared to other phenotypes. According to a study by Wang et al. [9], irrespective of metabolic health status, obesity was associated with lower GLS, but no significant decline in diastolic function.

In this study, the proportion of patients with Grade II diastolic dysfunction was higher in the MUO group compared to MHO and MUNO, whereas the proportion of patients with Grade I diastolic dysfunction was lower in the MUO group when compared to MHO and MUNO suggestive of the combined impact of metabolic health and obesity on the severity of diastolic dysfunction. A similar study by Lee et al. [8] found that obesity and metabolic health show a subclinical decline in systolic and diastolic function.

Low HDL was established to be an independent risk factor for CAD in various studies as HDL has antiatherogenic action by reverse cholesterol transport, reverses endothelial cell dysfunction, production of prostacyclin, helps vasodilation and anti-thrombotic action, inhibits apoptosis, decreases platelet aggregability and inhibiting LDL oxidation [10-15]. The findings of the present study align with existing literature by confirming that HDL is significantly affected in metabolically unhealthy individuals. In the MHO phenotype, among the five components of metabolic syndrome, maximum derangement was seen with HDL, followed by WC. In the MUNO phenotype, maximum derangement was seen in FBS, followed by HDL. In the MUO phenotype, maximum derangement was seen in TGs, followed by WC.

In this study, a multiple regression analysis showed that out of the seven variables correlated with systolic dysfunction, HDL, BMI, and FBS are significantly associated with systolic dysfunction. In contrast, TGs, WC, SBP, and DBP are not correlated with systolic dysfunction. A similar correlation was seen with diastolic dysfunction. Lee et al. [6] conducted a study to assess the effect of obesity and metabolic health status on LV structure and function and found that high SBP showed the greatest impact among the components of metabolic syndrome. Other studies also evaluated changes in metabolic parameters among MHO subjects. They compared them with metabolically healthy normal-weight subjects and found that MHO subjects showed higher metabolic derangement than MHNO. Individuals suggesting obesity impacts metabolic derangements [16-21]. The findings of the present study align with this existing literature, suggesting even in the absence of overt metabolic syndrome, can still negatively impact key metabolic parameters. This correlation underscores the need for careful monitoring of MHO individuals, as their metabolic status may deteriorate over time, increasing the risk of cardiovascular disease.

Limitations

This study assessed only the traditional metabolic variables as general risk factors in obesity phenotypes. It did not include other existing risk factors of CVD/CAD-like dietary factors, occupational stress, smoking, physical activity, surrogate sensitive lipid markers of CAD like apolipoprotein B100/apolipoprotein A-I ratio, and other obesity-related inflammatory adipokines like serum leptin, resistin, TNF. This study included 400 participants in four phenotype groups of 100 each. Age and sex matching was done. However, further categorization of groups into age categories could have yielded additional findings. Potential confounding factors (as for physical activity levels, dietary habits, and medications) which could influence the outcome were not considered in this study. The total number of patients was 100 in each group, which was our convenience but should be taken as statistical power. Last but not least, the limitation was the absence of collinearity assessment between predictors like BMI and WC.

## Conclusions

Although obesity and metabolic disease often overlap, there is variability in cardiovascular risk. Our findings indicate that obesity is linked to subclinical impairments in both systolic and diastolic function, regardless of metabolic disease status. Specifically, individuals in the MHO group showed worse global longitudinal strain, increased dyssynchrony, and early diastolic dysfunction compared to the control group. Obesity and poor metabolic health status were associated with decrement in systolic and diastolic function with increased cardiovascular morbidity and mortality. Among the components of metabolic syndrome, HDL and FBS were significantly correlated with systolic and diastolic dysfunction. BMI also significantly corresponded with systolic and diastolic dysfunction. The results of our study demonstrate an increased cardiovascular dysfunction in both obese and metabolically unhealthy populations.
